# Advances in the research of the role of macrophage/microglia polarization-mediated inflammatory response in spinal cord injury

**DOI:** 10.3389/fimmu.2022.1014013

**Published:** 2022-12-01

**Authors:** Sheng-Ping Fu, Si-Yu Chen, Qi-Ming Pang, Meng Zhang, Xiang-Chong Wu, Xue Wan, Wei-Hong Wan, Jun Ao, Tao Zhang

**Affiliations:** ^1^ Key Laboratory of Cell Engineering of Guizhou Province, Affiliated Hospital of Zunyi Medical University, Zunyi, Guizhou, China; ^2^ Department of Orthopaedic Surgery, Affiliated Hospital of Zunyi Medical University, Zunyi, Guizhou, China; ^3^ Collaborative Innovation Center of Chinese Ministry of Education, Zunyi Medical University, Zunyi, Guizhou, China; ^4^ The Clinical Stem Cell Research Institute, Affiliated Hospital of Zunyi Medical University, Zunyi, Guizhou, China

**Keywords:** macrophages, spinal cord injury, polarization, inflammatory response, miRNA, mesenchymal stem cells, microglia

## Abstract

It is often difficult to regain neurological function following spinal cord injury (SCI). Neuroinflammation is thought to be responsible for this failure. Regulating the inflammatory response post-SCI may contribute to the recovery of neurological function. Over the past few decades, studies have found that macrophages/microglia are one of the primary effector cells in the inflammatory response following SCI. Growing evidence has documented that macrophages/microglia are plastic cells that can polarize in response to microenvironmental signals into M1 and M2 macrophages/microglia. M1 produces pro-inflammatory cytokines to induce inflammation and worsen tissue damage, while M2 has anti-inflammatory activities in wound healing and tissue regeneration. Recent studies have indicated that the transition from the M1 to the M2 phenotype of macrophage/microglia supports the regression of inflammation and tissue repair. Here, we will review the role of the inflammatory response and macrophages/microglia in SCI and repair. In addition, we will discuss potential molecular mechanisms that induce macrophage/microglia polarization, with emphasis on neuroprotective therapies that modulate macrophage/microglia polarization, which will provide new insights into therapeutic strategies for SCI.

Introduction

Spinal cord injury (SCI) usually leads to permanent loss of motor, sensory and autonomic function below the site of injury (1). Unfortunately, about 1.3 million people worldwide suffer from SCI, with almost 180,000 new cases occurring yearly (2). Traumatic spinal cord injuries devastate the physical, financial, and psychological well-being of the injured person and their caregivers (3). The pathophysiological process of SCI can divide into primary and secondary injuries (4). Primary injury is irreversible, and the treatment of SCI is focused on minimizing the inflammatory response to secondary injuries (5, 6). To date, there is still a lack of effective treatment for neuroinflammatory conditions. Important neuroprotective measures presently applied in clinical practice include early surgical decompression, blood pressure augmentation, and intravenous glucocorticoids (GCs) (7, 8). However, these methods do not fundamentally improve the function of the injured spinal cord, and there is an urgent need to develop new therapeutic approaches to treat SCI.

As an important player in the immune response process, macrophages/microglia dominate the inflammatory process following SCI (9). After the injury, macrophages initially originated from resident activated-microglia and later primarily from circulating monocytes (10). As both infiltrating macrophages and resident microglia are derived from bone marrow mononuclear cells, they share similar molecular markers and functional characteristics, making it difficult to distinguish them at present (11). As a consequence, we use the term “macrophage/microglia” to describe these cells collectively. Studies have found that macrophage/microglia may have detrimental and beneficial roles in the injured spinal cord (12, 13). These seemingly contradictory functions of macrophages/microglia reflect the different phenotypes they acquire in response to different microenvironmental cues (14). Conventionally, macrophages/microglia are divided into two significant phenotypes: M1 and M2 (15). Classically activated macrophages/microglia (M1) is primed by Th1 cytokines, such as tumor necrosis factor-alpha (TNF-α) and interferon (IFN)-γ, and produce high levels of pro-inflammatory cytokines interleukin -6(IL-6), IL-23, IL-1β, and TNF-α (16, 17), which are pro-inflammation, induce axonal degeneration and cause neurotoxicity (18, 19). In contrast, alternatively activated macrophages/microglia (M2) are induced by Th2 cytokines such as IL-4 and IL-13 (17, 20). These cells secrete transforming growth factor-beta (TGF-β), IL-4, and IL-10 to suppress inflammation, induce angiogenesis, and promote tissue repair (20, 21). Growing evidence suggests that the transition of inflammatory macrophages/microglia to the M2 phenotype leads to reduced secondary damage and improved locomotor recovery after SCI (22, 23).

The current review will present and discuss the role of the inflammatory response and macrophages/microglia in SCI and repair. In addition, we will discuss potential molecular mechanisms that induce macrophage/microglia polarization, with emphasis on neuroprotective therapies that modulate macrophage/microglia polarization. It is hoped to provide direction and basis for future research on the mechanism of macrophage/microglia polarization and the treatment of SCI *via* regulating macrophages/microglia polarization.

The inflammatory response after SCI

Primary injury results from initial trauma to the spinal cord, which can cause rupture of the blood-spinal cord barrier (BSCB), bleeding, edema and oxidative damage (24, 25). This process initiates a secondary injury cascade that begins only a few hours after injury, leading to an inflammatory response, ischemia and progressive neuronal death (**Figure 1**) (25, 26). The BSCB consists of continuous endothelial cells, pericytes and glial cells with molecular junctions (27). Similar to the blood-brain barrier, the BSCB is critical for the exclusion of peripheral immune cells, various inflammatory and toxic metabolites from the CNS, which maintains microenvironmental stability (28, 29). Barrier integrity is compromised following SCI by disruption of interendothelial tight junctions (TJs) and adherent junctions (AJs) and by overall mechanical damage to the vasculature (30). The sustained permeability of the BSCB allows infiltration of peripheral inflammatory cells and factors into the spinal cord and leads to secondary injury (24). During the recovery stage, BSCB repair and glial scar formation limit the recruitment of “fresh” M2 macrophages/microglia to the injury site (14). This could also contribute to the persistence of inflammation after SCI.

**Figure 1:**
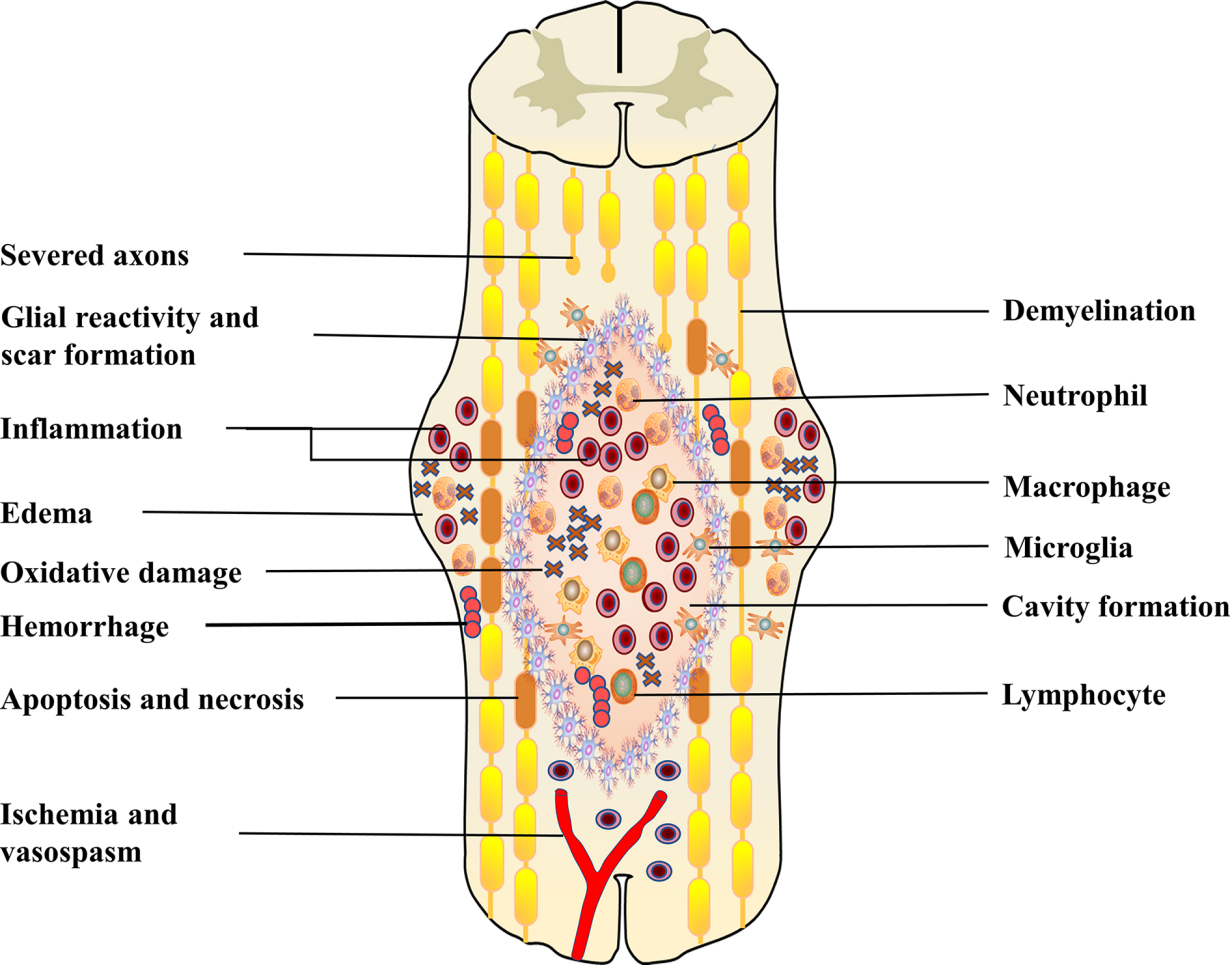
This figure shows the combination of pathophysiological events that occur post-SCI. This includes hemorrhage, oedema, inflammation, apoptosis, necrosis, oxidative damage, ischemia and vasospasm. Following primary injury, resident cells (astrocytes, microglia) are immediately activated and migrate to the site of injury. Subsequently, peripheral inflammatory cells, including neutrophils, macrophages and lymphocytes, infiltrate into the center of the damaged spinal cord. These activated immune cells can exacerbate the injury, causing neuronal death, which leads to axonal demyelination and disruption of synaptic transmission. In the subacute phase, fluid-filled cavities form in the center of the spinal cord. Astrocytes form a glial scar to isolate the damaged area. These sustained pathophysiological changes eventually lead to severe dysfunction below the damaged segment.

An inflammatory response is critical in the secondary injury cascade after SCI. It involves activated resident cells (microglia, astrocytes) and macrophages, neutrophils, and lymphocytes recruited from the peripheral blood to the site of injury (31, 32). These cells secrete pro-inflammatory cytokines, such as IL-1, IL-6, and TNF-α, all of which enhance the magnitude of the inflammatory response (4, 33). Damaged cells release damage-associated molecular patterns (DAMPs), such as small molecules and proteins, that induce sterile neuroinflammation after SCI (34, 35). DAMPs are perceived by pattern recognition receptors (PRRs), leading to the rapid activation of resident cells, such as microglia and astrocytes (36, 37). Activated microglia secrete various pro-inflammatory cytokines, including INF-γ, IL-6, TNF-α, and other cytotoxic factors (38, 39). At the same time, they were inducing vascular endothelial cells to express a variety of chemokines and cell adhesion molecules that contribute to the recruitment of peripheral immune cells to the site of injury (40, 41). As resident immune cells in the CNS, microglia also possess positive qualities. Fu et al. found that microglia may play a protective role after SCI by regulating astroglia scar formation, which insulates peripheral inflammatory cells in the core of the lesion, thus avoiding inflammation-mediated tissue damage (42). Similar to microglia, astrocyte membranes express a variety of PRRs, which can interact with DAMPs to initiate inflammatory responses (37, 43). Reactive astrocyte-derived permeability factor thymidine phosphorylase, which interacts with vascular endothelial growth factor A to induce blood-brain barrier disruption (44), alters the local microenvironment and facilitates the entry of peripheral immune cells into the CNS parenchyma. However, astrocytes are essential for regulating and alleviating inflammation after SCI and restoring microenvironmental homeostasis (45). First, the reactive astrocytes of the formed glial scar can constrain the harmful microenvironment (46). Second, astrocytes specifically eliminate glutamate, thereby reducing excitotoxic damage (46, 47). Neutrophils are the first peripheral immune cells to be recruited to a site of SCI (24, 48). These cells can promote phagocytosis and clearance of cellular debris and facilitate repair by secreting protease inhibitory factors (49, 50). However, infiltrating neutrophils release neutrophil extracellular traps (NETs), which subsequently promote BSCB disruption and neuroinflammation to exacerbate neuronal apoptosis and spinal cord edema following SCI (51). The endocytosis effect of macrophages on apoptotic neutrophils after tissue injury is the basis for eliminating inflammation and initiating tissue remodeling, which also plays an essential role in modulating neutrophil production (52, 53). Several studies have shown that macrophages are neurotoxic. For example, in the SCI model, macrophage depletion and lack of CX3CR1 signaling contributed to improved neurological function and tissue repair (54–56). In particular, macrophage depletion promoted recovery of TJ between vascular endothelial cells and reduced leakage of BSCB from the injured core post-SCI (57). These harmful or protective effects of macrophages on tissue regeneration are primarily owed to their different cellular phenotypes and the activation of specific intracellular signaling pathways. Like macrophages, activated lymphocytes have conflicting effects on SCI. On the one hand, T lymphocytes secrete various pro-inflammatory cytokines, such as IL-1β and INF-γ, resulting in neurological tissue damage (58). On the other hand, they could play an essential role in SCI and repair by regulating the function and recruitment of both innate and acquired immune cells following SCI (59). The conflicting results concerning the effects of T lymphocytes in restoration from SCI may be attributed to differences in the role of helper T-cell subsets (60).

Inflammation has both damaging and repairing effects. While the inflammatory response to SCI is beneficial and necessary for tissue repair following damage, sustained inflammation can be detrimental to tissue repair. As a result, effective suppression of excessive inflammation in SCI can improve the prognosis of SCI and promote nerve function recovery and tissue regeneration.

Role of macrophages/microglia in SCI

The inflammatory response following SCI leads to the infiltration of many inflammatory cells, and macrophages/microglia play a critical role in the evolution and development of inflammation.

Role of M1 macrophages/microglia in SCI

M1 macrophages/microglia can be induced by the Th1 cytokines such as TNF-α, IFN-γ, and lipopolysaccharide (LPS), which secrete destructive factors such as TNF-α, IL-1β, IL-6, and ROS (17, 61, 62), and can initiate the development of inflammation. Several studies have shown that the disruptive effects of activated macrophages/microglia are mainly ascribed to their M1 phenotype. For example, an increase in M1 polarized macrophages/microglia was observed in the context of SCI, which may inhibit M2 cells from repairing damaged tissue (63). Similarly, another study reported that Quercetin inhibits macrophages/microglia polarization towards the M1 phenotype *via* inhibition of the STAT1/NF-κB pathway, reduces neural tissue damage, and suppresses inflammatory response with a neuroprotective effect on SCI (64). macrophage/microglia have been demonstrated to contribute to spinal cord cavity formation and expansion after SCI. Fan et al. found that M1 macrophages/microglia may induce necroptosis in astrocytes following SCI through activation of TLR4/MyD88 signaling, which leads to enlargement of the spinal cord cavity (**Figure 2**) (65). These investigations suggest that M1 hinders neurogenesis and exacerbates secondary injury. However, M1 macrophages/microglia also possess positive qualities. These cells secrete soluble protein mediators such as vascular endothelial growth factor and enzymes that modify the extracellular matrix, which promote the proliferation of vascular endothelial cells (34). These seemingly conflicting results suggest that the M1 phenotype exerts some pro-tissue repair functions after all, such as clearance of cell debris soon after SCI. These findings suggest that future therapeutic strategies will need to inhibit M1 polarization at the correct time to improve the results of SCI.

**Figure 2:**
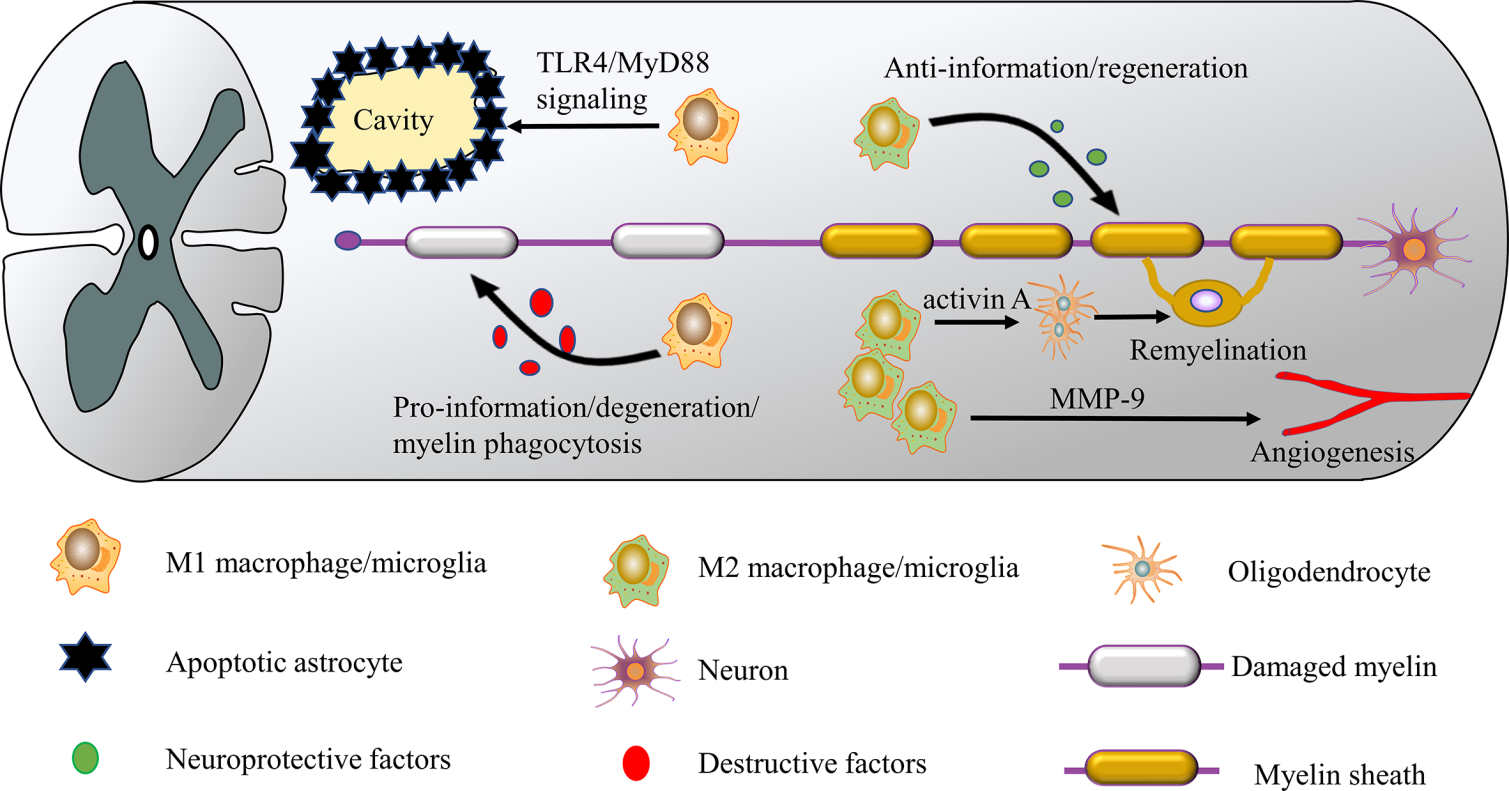
M1 and M2 macrophages/microglia have different roles in SCI. M1 macrophages/microglia are neurotoxic and release destructive factors that impair axon repair/regeneration. These cells can induce the necroptosis of astrocytes and promote the formation of the spinal cavity. In contrast, M2 macrophages/microglia have anti-inflammatory and neuroprotective effects, releasing neuroprotective factors such as IL-10 and TGF-β to improve SCI repair/regeneration. In addition, the M2 phenotype facilitates axon regeneration and angiogenesis by secreting activin A and matrix metalloproteinase 9 (MMP-9).

Role of M2 macrophages/microglia in SCI

M2 macrophages/microglia have anti-inflammatory functions and regulate tissue repair and remodeling, express CD206 (mannose receptor) and arginase (Arg)-1, and secrete anti-inflammatory cytokines such as IL-4 and TGF-β (62, 66). M2 macrophages/microglia remove cellular debris through phagocytosis, release many neuroprotective and trophic factors (14, 67), and produce anti-inflammatory cytokine IL-10, inducing the conversion of M1 macrophages/microglia to the M2 phenotype (68, 69), which plays a role in wound healing. In addition, M2 macrophages/microglia secret matrix metalloproteinase 9 (MMP-9) as a single tissue metalloproteinase 1 inhibitor, increasing their angiogenic potential (14, 70). Several studies have confirmed the neuroprotective role of the M2 phenotype in SCI. For example, in an SCI model, transplantation of M2 macrophages was effective in suppressing neuroinflammation and promoting functional recovery (71). Wang et al (22). Found that Butylphthalide plays an essential role in the anti-inflammatory response to SCI by promoting macrophages/microglia M2 polarization and inhibiting macrophages/microglia M1 polarization by activating the p38 pathway. In another study, azithromycin (AZM) was found to alter SCI macrophages/microglia polarization towards a beneficial M2 phenotype, thereby inhibiting the secondary injury process (23). Furthermore, M2 macrophages/microglia are an essential component of the CNS regenerative response, facilitating myelin regeneration by generating activin A to drive oligodendrocyte differentiation (**Figure 2**) (72). These studies suggest that M2 is crucial in suppressing inflammation and tissue repair. But it should be noted that excessive or prolonged M2 polarization may contribute to scar formation and fibrotic reactions that will hinder axonal regeneration and recovery of neurological function (73). Because M2 macrophages/microglia involved in wound healing also secrete large amounts of TGF-β, which subsequently activates fibroblasts, initiates and promotes the process of fibrosis (74, 75). Another potential concern is that long-term maintenance of the M2 phenotype may harm immune defenses and cause serious health consequences, such as tumor development (17). However, the dominance of M1 macrophages/microglia and the reduction in the number of M2 macrophages/microglia following SCI can expand tissue damage (76). Given the evidence in favor of a role for M2 macrophages/microglia in SCI anti-inflammatory and regeneration, M1-to-M2 phenotypic switches may create a favorable microenvironment for SCI repair.

Macrophages/microglia polarization

Macrophages/microglia polarization involves the interaction of various cytokines, chemokines, and transcription factors (**Figure 3**). Signaling molecules from the microenvironment bind specifically to receptors on the surface of the macrophages/microglia membrane and activate the corresponding signaling pathways and transcription factors, which initiate the macrophages/microglia polarization pathway (77, 78).

**Figure 3:**
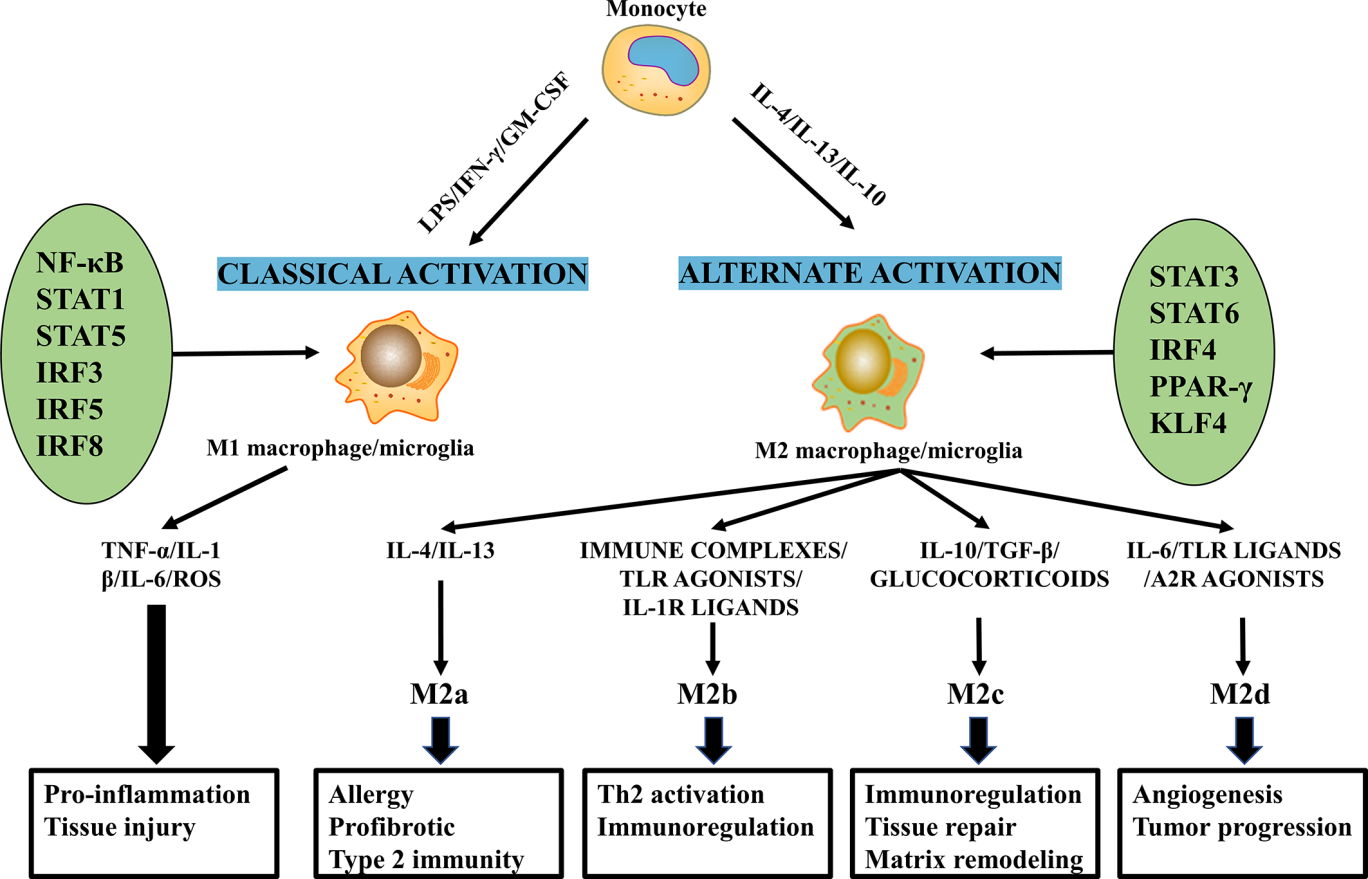
The molecular mechanisms of macrophages/microglia polarization and their functions.

Macrophages/microglia polarization to the M1 phenotype

Classically activated macrophages/microglia are activated by Th1 cytokines (IFN-γ, GM-CSF, and TNF-α) and lipopolysaccharide (LPS) (79, 80). Studies have shown that the TLR4/NF-κB pathway plays an essential role in M1 macrophages/microglia polarization (78, 81). For example, The LPS/TLR4 pathway activates NF-κB and interferon regulatory factor 3 (IRF3) to induce M1 polarization and facilitate the production of inflammatory factors (82). Members of the STAT family, including STAT1 and STAT3, are involved in regulating the M1 macrophages/microglia phenotype. For instance, IFN-γ binds to its receptor and activates Janus kinase, which phosphorylates STAT1, thereby inducing macrophages/microglia polarization to M1 (83). The roles of STAT3 are various as they are not only related to IL-6 induced M1 polarization but are also involved in IL-10 stimulated M2 polarization (84, 85). Notably, IL-17 enhances IFN-γM1-induced polarization by enhancing STAT1 phosphorylation while inhibiting IL-4-mediated M2 transformation by inhibiting STAT6 activation (86). To date, transcription factors, including NF-κB, STAT1, STAT5, IRF3, IRF5, and IRF8, have been shown to regulate transcription programs that control M1 macrophages/microglia polarizations (**Figure 3**) (85, 87–89).

Macrophages/microglia polarization to the M2 phenotype

The M2 phenotype can be primed by Th 2 cytokines, IL-13 and IL-4 (77, 90), as well as other factors, including IL-10, IL-33, M-CSF and TGF-β (77, 87, 91). Interestingly, IL-13 and IL-4 directly induce M2 macrophages/microglia activation, while other cytokines, such as IL-25 and IL-33, indirectly induce the M2 phenotype through the production of Th2 cytokines (92). STAT6 was the main transcription factor that induced M2 polarization (93, 94). IL-4 and IL-13 regulate the balance between M1/M2 macrophages/microglia in favor of the M2 phenotype *via* the IL-4Rα-STAT6 pathway (82, 95). We also found that IL-10 can polarize macrophages/microglia towards the anti-inflammatory M2 phenotype by activating the IL-10/STAT3 pathway (96). The importance of other transcription factors in phenotypic regulation has also been highlighted in several recent discoveries. For instance, IRF4 is an important transcription factor that controls M2 polarization (97, 98), and PPAR-γ and Kruppel-like factor 4(KLF4) control M2 polarization (99, 100).

M2 produce complex cytokines and have multiple functions, so they can be further subdivided into M2a, M2b, M2c, and M2d subtypes (**Figure 3**) (101). M2a, also known as wound healing macrophages/microglia, is induced by IL-4 and IL-13, express CD206 and C-C motif chemokine ligand 17 (CCL17), and secrete IL-10, TGF-β, and insulin-like growth factor (IGF) to promote tissue repair (102, 103). In addition, M2a macrophages/microglia are involved in tissue fibrosis, Th2 immune responses, and allergic reactions (78). Polarization of M2a macrophages/microglia promotes arginase 1 expression, restores axonal regeneration, promotes axonal regeneration and improves functional recovery post-SCI (104). M2b and M2c have similar immunomodulatory functions but different inducers. M2b macrophages/microglia are induced by immune complexes, Toll-like receptor (TLR) agonists or IL-1 receptor ligands, which produce both pro-inflammatory factors (IL-1β, IL-6 and TNF-α) and anti-inflammatory factor IL-10 (103), and play a role in Th2 activation and immune regulation (105, 106). In addition, M2b releases anti-inflammatory cytokines without causing neurotoxicity, facilitating recovery from SCI (107). M2c shows a regulatory phenotype induced by glucocorticoids, IL-10, and TGF-β exerts anti-inflammatory effects by secreting IL-10 and TGF-β (106, 108) and is associated with immunosuppressive behavior, matrix remodeling, and tissue repair (109, 110). Enhances M2b and M2c phenotypes. May coordinate the proliferative phase of wound healing and promote SCI repair (111). M2d macrophages/microglia, also called tumor-associated macrophages/microglia (TAM), are triggered by co-stimulation with TLR ligands and A2 adenosine receptor (A2R) agonists or IL-6 (103); these cells secrete IL-10, vascular endothelial growth factor (VEGF) and TGF-β (109), and contribute to angiogenesis and tumor metastasis (106).

Much knowledge of the phenotypic and functional characteristics of macrophages/microglia mentioned above is obtained from rodents. Notably, there are apparent differences between rodent macrophages/microglia and their human counterparts (112). For example, iNOS and Arg1 are mouse M1 and M2 markers, but are not expressed at significant levels in human macrophages/microglia at all (113, 114). Furthermore, macrophages/microglia responses to stimuli are variable between species, with GM-CSF and M-CSF being inducers of M1 and M2 polarization in mice but not in human macrophages/microglia (115). In several mouse tumor models, circulating monocytes are the major precursors of TAM (116, 117); in humans, in the context of bone marrow transplantation, lymphoma-associated macrophages/microglia were found to originate from bone marrow precursors (116), which suggests that human and mouse macrophages/microglia may differ in origin. More importantly, the M1/M2 dichotomy following macrophage/microglia polarization in animal models is not as evident in humans, and there is a continuum of activation states between M1 macrophages/microglia and M2 macrophages/microglia, the boundaries of which remain unclear (118). Some studies have found that human monocytes polarize to an M1 phenotype and M2 repair macrophages/microglia when microenvironmental conditions change, and vice versa (119, 120). This indicates that the phenotype and polarization mechanisms of human macrophages/microglia are much more complex than those of rodents. Consequently, a better understanding of the phenotypic and functional characteristics of human macrophages/microglia will provide a theoretical and research basis for the therapy of SCI.

Advances in neuroprotective therapies that regulate macrophages/microglia polarization in SCI

In healing wounds, the M1 to M2 macrophage/microglia phenotype transition supports the regression of inflammation and tissue repair (121). However, a similar M1 to M2 transition was not observed in spinal cord tissue repair process. Therefore, regulating macrophages/microglia to an anti-inflammatory phenotype is potentially a prospective therapeutic strategy for treating SCI.

Delivery of molecules to alter the macrophages/microglia phenotype

Several studies have demonstrated that inhibition of the M1 phenotype can be applied to the treatment of SCI, including blocking the activity of inflammatory cytokines and transcription factors involved in the induction of the phenotype. For example, in a mouse model of SCI, the blockade of IL-7 receptors further inhibits M1 polarization and promotes M2 polarization *via* down-regulating the expression of Th1 cytokines (IFN-γ, TNF-α) and up-regulating Th2 cytokines (IL-4, IL-13), which improved function following SCI (122). Similarly, blockade of IL-6 signaling promotes functional regeneration by inhibiting M1 and stimulating M2 macrophages/microglia activation post-SCI (123). In addition, Infliximab antagonism of TNF-α inhibited NF-κB activity, which is essential for M1 polarization (124), resulted in suppression of M1 polarization and improved motor function after acute SCI in rats (125). It was found that TNF-α levels increased shortly after SCI and that TNF-α activity had to be blocked immediately after injury to reduce the deleterious effects induced by TNF-α (126). Because delayed blockade of TNF-α activity do not effectively suppress M1 activation, which has no effect on repair following SCI (127). Finally, Nanoparticle delivery of siRNA silences the transcription factor IRF5, which downregulates M1 macrophage/microglia-related gene expression, resulting in a dramatic reduction in M1 macrophages/microglia numbers and a significant increase in the number of M2 macrophages/microglia in the wound, reducing neuroinflammation, inhibiting demyelination and promoting wound healing (128).

Numerous studies have shown that in addition to supplying M1-inhibiting molecules, releasing M2-promoting molecules can also promote tissue repair. For instance, systemic or intraspinal administration of IL-4 after SCI increased levels of the anti-inflammatory cytokine IL-10 and promoted M2 replacement activation of macrophages/microglia (129, 130). Regardless of the route of administration, IL-4 treatment significantly reduced the expression of the inflammatory marker nitric oxide synthase (iNOS) (129, 130). Similarly, sustained delivery of IL-10 improved neurological function post-SCI, which induces conversion of macrophages/microglia to an anti-inflammatory M2 phenotype and reduces the inflammatory response following SCI with depression of TNF-α and IL-1β production (68). In addition, IL-10 upregulates anti-apoptotic factors such as B-cell lymphoma 2 (Bcl-2), which exhibits direct trophic effects on neurons and improves the neurotoxic microenvironment (131).

Peroxisome proliferator-activated receptor gamma (PPAR-γ) is a ligand-dependent nuclear receptor that regulates the immune-inflammatory response (132). As we mentioned earlier, PPAR-γ plays an important role in macrophages/microglia polarization. Several studies have revealed that PPAR-γ activation-induced anti-inflammatory effects are associated with macrophages/microglia polarization (133, 134). activation of PPAR-γ induces macrophages/microglia polarization towards the M2 subtype, thus exerting its beneficial anti-inflammatory effects (135). Importantly, PPAR-γ is a broadly distributed nuclear receptor whose activation has caused to a reduction in the pro-inflammatory cascade in various CNS diseases (136). Because activation of PPAR-γ in CNS diseases suppresses the expression of proinflammatory cytokines TNF-α, IL-1β and iNOS, increases neuronal survival (137, 138). For example, the PPAR-γ agonist thiazolidinediones (TZDs) inhibited the inflammatory cascade and increased tissue retention, thereby improving motility in a rodent model of SCI (139, 140), and TZDs effectively reduced the release of pro-inflammatory factors such as IL-1β, IL-6 and TNF-α from macrophages/microglia (136). It is clear that PPAR-γ activation plays an important anti-inflammatory role in SCI. However, to date, there is very little direct *in vivo* evidence demonstrate that PPAR-γ activation improves anatomical and motor recovery after SCI through promoting the polarization of M2 macrophages/microglia.

Mesenchymal stem cell and exosome therapy

The therapeutic effect of MSCs on SCI may be partly attributed to the transplantation-induced polarization of macrophages/microglia towards an anti-inflammatory phenotype (141). Because MSCs have immunomodulatory properties and secrete immunomodulatory factors such as IL-10, TGF-β, IDO, TNF-inducible gene-6 (TSG-6) and prostaglandin-E2 (PGE2), these cytokines induce polarization of macrophages/microglia towards the M2 phenotype (96, 142–145), thereby inhibiting inflammation leading to remodeling and disease healing effects. Our previous study found that PBMSCs effectively induced macrophages/microglia polarization towards the M2 phenotype, upregulated the expression of M2 surface markers (Arg-1, CD206) and cytokines (IL-10, CCL22, TGF-β1), while significantly inhibiting the production of IL-1β, IL-6 and TNF-α, and promoted functional recovery after spinal cord injury (146, 147). In the acute phase of SCI, MSC transplantation regulated macrophages/microglia polarization from M1 to M2 cells, promoted the expression of anti-inflammatory mediators IL-4 and IL-13, inhibited the production of pro-inflammatory mediators TNF-α and IL-6, and suppressed scar formation and neuronal demyelination, providing a permissive environment for axonal extension and functional recovery (148, 149).

Although MSCs have great potential to treat SCI, the poor microenvironment after injury is not conducive to MSCs survival, which hinders their further clinical application. Therefore, any approach to improve the biological activity of transplanted MSCs *in vitro* and *in vivo* is of great value. Notably, pretreated MSCs show better immunomodulatory capacity, enhanced cell survival and homing ability, resulting in more effective repair of damaged spinal cord (150, 151). A number of pretreatment methods have been explored to enhance MSC therapeutic capacity, including hypoxia, inflammatory cytokines, genetic modifications and 3D culture (150, 152–154). For example, MSCs cultured under hypoxic conditions secrete more cytokines and growth factors, resulting in enhanced therapeutic function (155, 156). *In vivo*, hypoxic preconditioning (HP) improves the survival and migration of MSCs and enhances their therapeutic potential against SCI (157). It was found that stimulation of MSCs with IL-1β and IFN-γ enhanced their ability to induce macrophages/microglia polarization to the M2 phenotype and immunomodulatory functions (158, 159). TNF-α was also used as a pretreatment for MSCs, and TNF-α pretreatment exhibited anti-inflammatory effects by promoting the secretion of immunomodulatory factors (such as IDO, PGE2 and HGF) by MSCs (160). Genetic modifications have been reported to improve the efficacy of MSCs. Basic fibroblast growth factor overexpressing MSCs significantly improved treatment outcomes, for example, reducing glial scar formation, improving nerve regeneration and endogenous neural stem cell (NSC) proliferation, and increasing recovery of motor function in the hind limb (161). Similarly, MSCs transplanted with IL-10 overexpressing cells were more capable of inducing M2 type macrophages/microglia, significantly improving functional recovery and reducing lesion size and demyelination area after SCI (162). 3D culture could be another unique method to further improve the immunomodulatory capacity of MSCs. Compared to 2D culture, MSCs in 3D culture were able to secrete high yields of highly productive and active exosomes (163). Han et al (164). found that bone marrow-derived mesenchymal stem cells cultured in 3D collagen scaffolds exhibited unique characteristics, including significantly reduced *in vitro* LPS-induced macrophage/microglia activation and enhanced neurotrophic factor secretagogues. These results suggest that pretreatment enhances the biological function of MSCs *in vivo* and *in vitro* and could help them adapt to the new transplant microenvironment.

Recent studies have found that the therapeutic effects of MSCs are largely attributable to exosomes (165). Exosomes are important paracrine factors that can be used as direct therapeutic agents (166). They not only modulate the immune response and suppress inflammation, promote axonal regeneration and angiogenesis, but also play an important role in the repair of SCI by inhibiting apoptosis as well as maintaining the integrity of the blood-spinal cord barrier (166–169). Several studies have confirmed the therapeutic effect of MSC-exosomes in relation to the induction of macrophages/microglia polarization (76). For example, BMSCs-derived exosome miR-124-3p attenuated spinal cord ischemia-reperfusion injury (SCIRI)-induced tissue damage and nerve injury by silencing endoplasmic reticulum-to-nucleus signaling 1 leading to polarization of macrophages/microglia towards M2 and inhibiting apoptosis (170). Sun et al (165). found that human umbilical cord mesenchymal stem cells exocytosis triggered the polarization of macrophages/microglia from M1 to M2 phenotype and improved functional recovery after SCI by downregulating inflammatory cytokines such as IL-6, TNF-α, and IFN-γ. Subsequently, MSC-derived exosomes exhibited neuroprotective effects by altering the M1/M2 macrophages/microglia ratio and shifting the balance towards the M2 phenotype (171, 172). Compared to MSCs, MSC-derived exosome therapy avoids the problems of low MSC survival and the potential differentiation of MSCs into other cell types (172). In addition, exosomes are more stable, safer and less immunogenic (168). Therefore, MSC-derived exosomes may be a promising therapeutic strategy for the treatment of SCI.

MicroRNAs regulate macrophages/microglia polarization

A large number of microRNAs (miRNAs) have been identified in the mammalian central nervous system and play an important role in neural regeneration, genesis and development (3, 173). miRNAs are a class of small non-coding RNAs that act as transcriptional regulators, which are involved in the pathophysiological processes of SCI and are considered to be effective therapeutics for SCI (174, 175). Some investigators have found that miR-155 deletion not only reduces macrophages/microglia-mediated neurotoxicity and increases neuroprotection, but also improves functional recovery and reduces neuropathic pain (176, 177). miR-182 has inhibitory effects on the inflammatory response to various types of injury, and in a mouse model of SCI, miR-182 ameliorates the secondary injury after SCI by blocking the IKKβ/NF-κB pathway to inhibit apoptosis and inflammatory responses (178). Similarly, miR-494 is important in regulating the onset, progression and repair of SCI; Huang et al. Showed that miR-494-modified exosomes delivered to the diseased spinal cord improved the local immune environment, inhibited neuronal apoptosis and the release of pro-inflammatory factors, thereby promoting neurofilament regeneration and recovery of behavioral function (3). This evidence from the literature suggests that miRNAs are modulators of secondary injury and repair after SCI and a novel intervention pathway to improve recovery after SCI.

Recent studies have shown that miRNAs control macrophage/microglia differentiation and activity by regulating the signaling of key transcription factors (179, 180), which subsequently influence the progression of inflammation. The main transcription factors involved in regulating macrophages/microglia polarization include signal transducers and activators of transcription (STATs), KLF, interferon regulatory factors (IRFs), PPAR, C-MYC, and C/EBPs (**Figure 4**) (179, 181). miR-146b, for example, targets the transcription factor IRF5 to significantly inhibit M1 polarization and improve the development of colitis (182). miR-155 reduced myocardial inflammation and improved cardiac function by increasing STAT6 phosphorylation that promoted M2 but prevented M1 polarization (183). Subsequently, miR-125a negatively regulated IRF5, promoting M2 polarization and improving inflammation after SCI (184). Another study revealed that miR-22-3p reduced ischemia/reperfusion (I/R) injury in the spinal cord by inhibiting IRF5 to promote macrophages/microglia shift to the M2 phenotype and suppress tissue inflammation (185). Interestingly, multiple miRNAs can target the same transcription factor, each with a different target. For example, miR-27a, miR-130a and miR-130b promote M1 polarization by inhibiting PPAR-γ expression (186–188). In contrast, miR-124 can target different transcription factors, such as STAT3 and C/EBP-α, respectively, to promote M2 polarization (189, 190). To date, accumulating evidence confirms that miR-27a (186), miR-27b (191), miR-130a (187), miR-155 (183, 192), miR-21 (193), miRNA-125b (194) and miRNA-26a (195, 196) have been shown to promote M1 polarization, whereas miR-146b (197), miR-223 (198), miR-93 (199), miR-124 (189), and let-7c (200) induce M2 macrophages/microglia polarization *via* targeting various transcription factors and adapter proteins (**Figure 4**). These miRNAs regulate the transcriptional expression of target genes by targeting transcription factors, which determine the functional polarization of macrophages/microglia.

**Figure 4:**
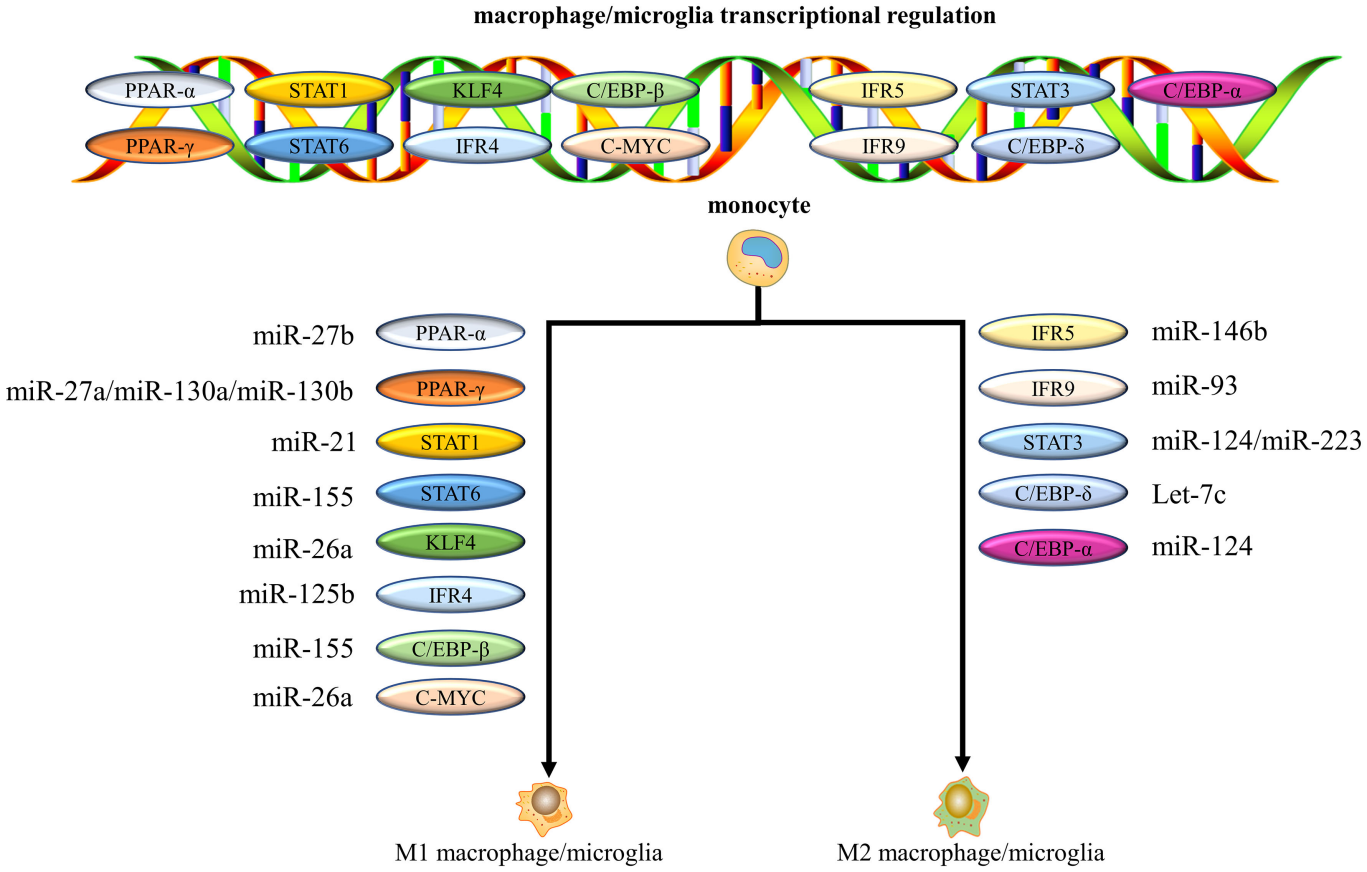
miRNAs control macrophages/microglia polarization by regulating the signaling of key transcription factors. Multiple miRNAs can target the same transcription factor, and each miRNA can have a different target.

The mechanisms by which miRNAs regulate macrophage/microglia polarization *via* transcription factors appear complex. For example, it has been demonstrated that in adipocytes miR-34a promotes M1 and inhibits M2 polarization by directly targeting KLF4 (100, 201). However, it has also been shown that miR-34a can inhibit pro-inflammatory macrophages/microglia polarization and enhance anti-inflammatory macrophages/microglia phenotypes (181, 202). The reasons behind the conflicting effects of miR-34a on macrophage/microglia polarization are unclear. These may be attributed to the specificity of miRNAs for different tissues and cell types. And each miRNA can have different targets and multiple miRNAs can have the same target gene (203, 204). This may result in miR-34a having different effects on macrophage/microglia polarization. Understanding the molecular mechanisms by which miRNAs regulate macrophages/microglia can help provide the basis for macrophages/microglia-centric therapeutic strategies.

M2 macrophages/microglia adoptive immunotherapy

M1 macrophages/microglia and anti-inflammatory M2 macrophages/microglia were detected early in the local microenvironment after SCI injury, but 1 week after injury, M1 was predominant and M2 was minimal (205). It has been found that using bone marrow-derived M2 adoptive transfer 7 days after SCI increases the proportion of M2 macrophages/microglia in the injured spinal cord and has a neuroprotective effect (206, 207). The increase in the number of M2, through the production of anti-inflammatory cytokines such as IL-10 and TGF-β, shifts the immune response from a Th1-dominant to a Th2-dominant one, induces more local macrophages/microglia polarization to the M2 phenotype, and creates a local microenvironment conducive to the protection of neuronal function and inhibition of demyelination (206). Chen et al. found that M2 macrophages/microglia adoptive immunity can reduce the expression of genes associated with inflammatory signaling pathways such as antigen processing and presentation, phagosomes, cell adhesion molecules, cell-mediated cytotoxic natural killing, endocytosis, and proteasomal and Toll-like receptor signaling pathways (207). These could explain the mechanism by which M2 adoptive immunotherapy provides neuroprotection against SCI. However, the specific mechanisms of adoptive immunotherapy remain to be investigated in depth.

Conclusion

SCI causes complex pathophysiological changes that are fundamental to the pathogenesis of secondary injury. Among all changes, inflammation is the main obstacle to neurological recovery and directly influences disease progression. Macrophages/microglia are one of the primary cells involved in neuroinflammation in SCI. In response to damaged spinal cord microenvironment signals, macrophages/microglia can polarize into pro-inflammatory (M1) and anti-inflammatory (M2) macrophages/microglia. In recent years, many studies have demonstrated that the transition of inflammatory macrophages/microglia to the M2 phenotype leads to a reduced secondary injury and recovery of motor capacity following SCI. Based on insights into the effects of macrophages/microglia and their polarization on SCI and repair, several neuroprotective therapies targeting macrophages/microglia polarization in SCI have been investigated. These approaches have contributed to neural tissue regeneration and functional recovery post-SCI. However, the therapeutic approach to inflammation in SCI should switch from broad promoting macrophages/microglia polarization towards M2 to subtle regulation of the balance among their phenotypes. As mentioned above, excessive accumulation of any one phenotype of macrophages/microglia is detrimental. The accumulation of large numbers of M1 macrophages/microglia results in chronic inflammation and tissue destruction; at the same time, excessive or prolonged M2 polarization can promote scar tissue proliferation that will impede axonal regeneration. As a result, we must carefully enhance the correct phenotype at the right time to effect neuroinflammation regression, reduce secondary damage and promote functional recovery.

## Data availability statement

The original contributions presented in the study are included in the article/supplementary material Further inquiries can be directed to the corresponding author.

## Author contributions

S-PF wrote the manuscript. S-YC, Q-MP, MZ, X-CW, XW, W-HW, JA, and TZ edited the paper. All authors contributed to the article and approved the submitted version.

## Funding

This work was supported by grants from the National Natural Science Foundation of China (No.81960299), the Guizhou Provincical Science and Technology Foundation (No.[2020]1Y324).

## Conflict of interest

The authors declare that the research was conducted in the absence of any commercial or financial relationships that could be construed as a potential conflict of interest.

## Publisher’s note

All claims expressed in this article are solely those of the authors and do not necessarily represent those of their affiliated organizations, or those of the publisher, the editors and the reviewers. Any product that may be evaluated in this article, or claim that may be made by its manufacturer, is not guaranteed or endorsed by the publisher.
